# The *Netherlands Heart Journal*: special issue on COVID-19

**DOI:** 10.1007/s12471-020-01482-2

**Published:** 2020-07-16

**Authors:** J. Vendrik, J. J. Piek

**Affiliations:** Department of Cardiology, Amsterdam UMC, Heart Centre, University of Amsterdam, Amsterdam, The Netherlands

The ongoing coronavirus disease 2019 (COVID-19) pandemic has had a huge impact on our personal and professional life alike. In this special issue of the *Netherlands Heart Journal* focussing on COVID-19, several aspects of the pandemic influencing our profession are highlighted.

Starting with a broader overview, de Vries [1] very thoroughly describes the severe acute respiratory syndrome coronavirus 2 (SARS-CoV-2) life cycle, its transmission and the clinical and immunological features of COVID-19. Different approaches to developing a COVID-19 vaccine are touched upon and the perspectives for treating COVID-19 with antiviral drugs, immunomodulatory agents and anticoagulants/antithrombotics are described. Several of the aforementioned categories of medication may influence the cardiovascular system directly, hence influencing the care and treatment of our cardiac patients. van den Broek et al. [2] describe the consequences of using chloroquine, a potential therapeutic option in COVID-19. They show a significant and clinically relevant prolongation of the QTc interval in chloroquine-treated patients, highlighting the need for intensive ECG monitoring in COVID-19 patients. The article of Sinkeler et al. [3] draws the same conclusion from an even larger cohort of COVID-19 patients, whilst adding that since the computer-based algorithm for calculating the QTc interval seems to overestimate the actual QTc, manual QTc measurements are advisable before adjusting chloroquine doses or even withdrawing chloroquine treatment. Reviewing this, Wilde and Offerhaus [4] comment on the use of hydroxychloroquine and chloroquine and refer to them as ‘the president’s drug’. They also discuss how to assess the efficacy and safety of these medications in the current pandemic.

Opposed to the hypothesis that potential therapies for COVID-19 affect the cardiovascular system, de Vries [5] describes the influence of blood-pressure-lowering drugs on the manifestation of SARS-CoV‑2. In this Point of View paper, currently available evidence is summarised regarding the use of angiotensin-converting enzyme inhibitors (ACEIs) and angiotensin II type‑1 receptor blockers (ARBs) during this pandemic. de Vries concludes that, although further research on the influence of blood-pressure-lowering drugs, including those not targeting the renin-angiotensin system, is warranted, there are presently no compelling clinical data showing that ACEIs and ARBs increase the likelihood of contracting COVID-19 or worsen the outcome of SARS-CoV‑2 infections. At the moment it appears that the pathophysiology of COVID-19 seems to predominantly affect the respiratory system. van den Heuvel et al. [6] describe the cardiac involvement in hospitalised COVID-19 patients, as they frequently display elevated cardiac biomarkers. They found no relation between elevated troponin T or N‑terminal pro-brain natriuretic peptide levels and left or right ventricular function, while adding that echocardiography is of limited value in screening for ventricular failure in hospitalised COVID-19 patients.

COVID-19 has had a huge impact on cardiac care in recent months, and the article of Mayol et al. [7] delivers undeniable evidence of this. The authors describe the results from a telematic survey for all Latin American countries, showing a significant reduction in cardiac care activity during the COVID-19 pandemic, including a significant reduction in care for ST-elevation myocardial infarction (STEMI). As this almost certainly has a negative impact on STEMI-associated mortality and morbidity, they state that healthcare providers must find a way to alert and inform patients about the suspected symptoms of STEMI, and emphasise the need for patients to call for emergency care, in order to ensure a timely diagnosis and reperfusion treatment.

Further alterations in daily clinical practice during and after the COVID-19 pandemic are proposed in this issue. Kemps et al. [8] provide us with practical recommendations for continuing to provide cardiac rehabilitation services. Current public health measures and reorganisations in outpatient cardiac care render traditional centre-based cardiac rehabilitation almost impossible. In addition, they describe the potential negative influence of public health measures on lifestyle behaviour and general well-being. As we need to find a way to continue regular cardiac care amidst this crisis, Vendrik et al. [9] propose a means of enabling ongoing transfemoral transcatheter aortic valve implantation (TF-TAVI). TF-TAVI in current practice is predominantly performed as an elective catheterisation laboratory procedure, and the COVID-19 crisis has created a relative unavailability of anaesthesiological support. The authors describe the performance of TF-TAVI with a dedicated nurse performing local analgesia instead of an anaesthesiologist as feasible and safe in a selected group of patients. Continuing to perform TAVI procedures in this way may theoretically prevent non-COVID-related deaths. Finally, limited evidence regarding cardiovascular complications and long-term outcomes of COVID-19 in (elite) athletes and non-hospitalised patients is available. Verwoert et al. [10] provide us with a practical guide to pre-participation screening, diagnostic and therapeutic strategies after COVID-19 in athletes, as less stringent restrictions for sports and exercise will soon be employed.

We hope that the readers of this journal find this information valuable for their care of cardiac patients in these unusual circumstances during the COVID-19 pandemic.

## References

[1] de Vries AAF (2020). SARS-CoV-2/COVID-19: a primer for cardiologists. Neth Heart J.

[2] van den Broek MPH, Möhlmann JE, Abeln BGS, Liebregts M, van Dijk VF, van de Garde EMW (2020). Chloroquine-induced QTc prolongation in COVID-19 patients. Neth Heart J.

[3] Sinkeler FS, Berger FA, Muntinga HJ, Jansen MMPM (2020). The risk of QTc-interval prolongation in COVID-19 patients treated with chloroquine. Neth Heart J.

[4] Wilde AAM, Offerhaus JA (2020). The ‘president’s drug’. Neth Heart J.

[5] de Vries AAF (2020). Renin-angiotensin system inhibition in COVID-19 patients. Neth Heart J.

[6] van den Heuvel FMA, Vos JL, Koop Y (2020). Cardiac function in relation to myocardial injury in hospitalised patients with COVID-19. Neth Heart J.

[7] Mayol J, Artucio C, Batista I (2020). An international survey in Latin America on the practice of interventional cardiology during the COVID-19 pandemic, with a particular focus on myocardial infarction. Neth Heart J.

[8] Kemps HMC, Brouwers RWM, Cramer MJ, Jorstad HT, de Kluiver EP, Kraaijenhagen RA (2020). Recommendations on how to provide cardiac rehabilitation services during the COVID-19 pandemic. Neth Heart J.

[9] Vendrik J, de Boer J, Zwiers W (2020). Ongoing transcatheter aortic valve implantation (TAVI) practice amidst a global COVID-19 crisis: nurse-led analgesia for transfemoral TAVI. Neth Heart J.

[10] Verwoert GC, de Vries ST, Bijsterveld N (2020). Return to sports after COVID-19: a position paper of the Dutch Sports Cardiology Section of the Netherlands Society of Cardiology. Neth Heart J.

